# Why Do Some First Nations Communities Have Safe Water and Others Not? Socioeconomic Determinants of Drinking Water Risk

**DOI:** 10.5539/gjhs.v8n9p99

**Published:** 2015-10-29

**Authors:** Brandon Brown, Renata Wachowiak-Smolíková, Nicholas D. Spence, Mark P. Wachowiak, Dan F. Walters

**Affiliations:** 1 Department of Geography, Nipissing University, North Bay, Canada; 2 Department of Computer Science and Mathematics, Nipissing University, North Bay, Canada; 3 Department of Pediatrics, University of Alberta, Edmonton, Canada

**Keywords:** Canada, public policy, socioeconomic factors, vulnerable populations, water

## Abstract

Securing safe and adequate drinking water is an ongoing issue for many Canadian First Nations communities despite nearly 15 years of reports, studies, policy changes, financial commitments, and regulations. The federal drinking water evaluation scheme is narrowly scoped, ignoring community level social factors, which may play a role in access to safe water in First Nations. This research used the 2006 Aboriginal Affairs and Northern Development Canada First Nations Drinking Water System Risk Survey data and the Community Well-Being Index, including labour force, education, housing, and income, from the 2006 Census. Bivariate analysis was conducted using the Spearman’s correlation, Kendall’s tau correlation, and Pearson’s correlation. Multivariable analysis was conducted using an ordinal (proportional or cumulative odds) regression model. Results showed that the regression model was significant. Community socioeconomic indicators had no relationship with drinking water risk characterization in both the bivariate and multivariable models, with the sole exception of labour force, which had a significantly positive effect on drinking water risk rankings. Socioeconomic factors were not important in explaining access to safe drinking water in First Nations communities. Improvements in the quality of safe water data as well as an examination of other community processes are required to address this pressing policy issue.

## 1. Introduction

Why do some First Nations communities have safe drinking water and others not? This is a vexing question that has been the subject of great inquiry (White, Murphy, & Spence, 2012; White, 2012; Walters, Spence, Kuikman, & Singh, 2012; Spence & Walters, 2012; LaBoucane-Benson, Gibson, Benson, & Miller, 2012; Neegan Burnside Ltd., 2011; Swain, Louttit, & Hrudey, 2006; Indian and Northern Affairs Canada, 2003). The Canadian federal government uses a risk-based approach to assess and manage health threats to First Nations drinking water systems. Aboriginal Affairs and Northern Development Canada (AANDC) has been tracking the risk level (i.e., low, medium, high) of First Nations drinking water systems since 2001. The *Risk Evaluation Guidelines* assess the overall risk score using criteria in five categories (source water, design, operation, reporting and operator) (Indian and Northern Affairs Canada, Public Works and Government Service Canada, Environment Canada, Health Canada, 2005). A detailed description of the survey questions and risk categories is provided elsewhere (Walters, Spence, Kuikman, & Singh, 2012). The risk evaluations are used by AANDC to minimize health risks by addressing specific community threats, and to develop a national strategy. However the empirical evidence indicates that the federal policies and financial commitments over the past decade have not reduced the total number of high risk drinking water systems in First Nations inquiry (White, Murphy, & Spence, 2012). Thus, what is driving access to safe drinking water?

**Figure 1:**
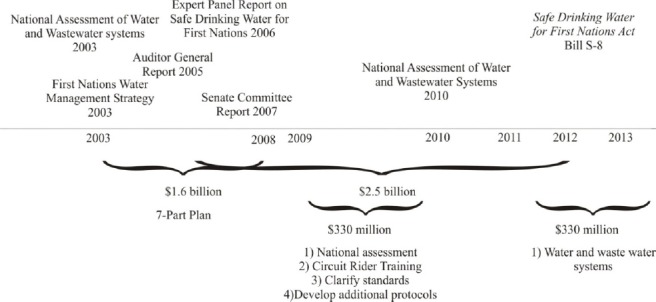
Federal policy, financial and legislative response to first nations water and wastewater challenges

The federal government has responded to drinking water threats by announcing new policies and financial commitments to enhance the capacity of First Nations to deliver safe drinking water (Figure 1). In 2003, the federal government revealed a five-year plan and $1.6 billion to improve drinking water in First Nations communities. The First Nation Water Management Strategy (FNWMS) involved a seven-part plan to reduce the risk of unsafe or inadequate drinking water needs. Additional funding of $330 million in 2009 and 2011 helped to extend implementation of the FNWMS. Following the evacuation of community members from Kashechewan because of E. coli contamination, the government announced $2.5 billion to help construct, maintain or operate First Nations water and wastewater systems. In 2013, the Federal government assented the *Safe Drinking Water in First Nations Act*. However, current efforts appear to be inadequate to deal with the complexity of the problem.

There have been three National Assessments of Water and Wastewater Systems (i.e. 2003, 2006, 2010) in First Nations communities (Neegan Burnside Ltd., 2011; Swain, Louttit, & Hrudey, 2006; Indian and Northern Affairs Canada, 2003). Empirical evidence indicates that the number of high risk drinking water systems decreased from 218 in 2003 to 178 in 2006. However, the number of high risk drinking water systems increased to 314 in 2010 (Table 1). By only reporting the national summary data, it is impossible to ascertain whether the high risk communities in 2010 were in the low or medium risk categories in either 2003 or 2006.

**Table 1:** Summary of national risk evaluation survey results

Drinking Water Risk Level	National Risk Evaluation Summary Data (%)		

	2003 (*n* = 740)	2006 (*n* = 739)	2010 (*n* = 807)
Low Risk	185 (25)	263 (36)	215 (27)
Medium Risk	337 (46)	298 (40)	278 (34)
High Risk	218 (29)	178 (24)	314 (39)

Source: Indian and Northern Affairs Canada Drinking Water Surveys, 2003, 2006, 2010.

Other government reports cite that the regulatory framework; location; accountability; costs and financing; training and management capacity; operators; self-governance; technical standards; and population growth as contributing to the drinking water risk level of First Nation communities (Neegan Burnside Ltd., 2011; Swain, Louttit, & Hrudey, 2006; Auditor General of Canada, 2005; Auditor General of Canada, 2011).

The academic literature has also engaged this issue from a variety of perspectives, emphasizing some of the underlying or “upstream” factors contributing to this complex problem, such as colonial history, policy processes (participation and consultation), culture, indigenous knowledge, self-determination, political power, community capacity (human, physical and social capital), perception of health risks, technological capacity, adaptive sustainability, and source water protections (White, Murphy, & Spence, 2012; Swain, Louttit, & Hrudey, 2006; von der Porten & de Loe, 2010; Morales, 2006; Reading et al., 2011; Patrick, 2011). A common thread in these works is that community vulnerability and associated process at this level of analysis may contribute to understanding and be an effective point for interventions to secure safe water. That being said, there is a lack of empirical assessments at the national level examining the community processes associated with safe water, reflecting a lack of data, and a focus on small-scale case studies.

The federal drinking water evaluation scheme is too narrowly scoped to capture other possible threats to safe water in First Nations. The federal drinking water system evaluation scheme is skewed towards the technological threats (Walters, Spence, Kuikman, & Singh, 2012). As such, there is an incentive to focus on the technical threats to reduce risk, with less emphasis on the human, financial and social aspects (White, Murphy, & Spence, 2012). The current work explores relationships between overall drinking water system risk and community well-being, with a focus on socioeconomic factors (i.e., labour force, education, income, housing) at the community scale. Although the measure of communities is far from exhaustive, focusing on community socioeconomic factors provides a starting point to assess how capacity may be associated with the ability to access safe drinking water. The socioeconomic vulnerability of communities is related to the availability of safe water in number of ways (White, Murphy, & Spence, 2012). The framework for the provision of safe water sets the stage in terms of how community well-being may influence safe water. Communities that possess the capacity to provide the human and financial resources to effectively address and manage issues, including the water and wastewater systems, would be better positioned to yield positive outcomes. Therefore, we hypothesize a negative association between drinking water system risk and community socioeconomic factors. This type of analysis has not been done at a large scale level, and offers a possible new approach for understanding the issue by enhancing our scientific understanding of the processes contributing to safe water, and ways for the federal government to respond to First Nations’ drinking water health risks.

## 2. Methods

### 2.1 Measures

#### 2.1.1 Community Well-Being

This work used the Community Well-Being Index (CWB) dataset from the year 2006, obtained through a strategic research partnership with Aboriginal Affairs and Northern Development Canada. Using 2006 Census of Canada data, communities were defined as census subdivisions, including municipalities and equivalent geographical spaces, such as Indian reserves, Indian settlements, and unorganized territories. There are some restictions with accessing community scale data; only data from communities with a minimum population of 65 and where the percentage of required responses left unanswered by respondents was not over 25% was used. This analysis was focused on the population of First Nations communities or “reserves” in Canada, as defined by Aboriginal Affairs and Northern Development Canada and Statistics Canada, which included all census subdivisions with a legal affiliation with Indian Bands, in addition to select census subdivisions in Northern Saskatchewan, the Northwest Territories, and the Yukon Territory. In terms of population coverage of Aboriginal peoples in the 2006 Census, there were 22 incompletely enumerated Indian reserves and Indian settlements (Statistics Canada, 2009).

The CWB is a composite measure derived from four indicators, including education, labour force activity, income, and housing. Education is composed of the proportion of the community with “high school plus” and “university.” Labour force measures labour force participation and the employment rate. Income is captured by income per capita. The housing measure captures both the quantity and quality of housing: percentage of the population living in dwellings that contain no more than one person per room and percentage of the population living in dwellings that are not in need of major repairs. The CWB and its four indicators range from zero to one hundred, with higher numbers representing greater well-being. Further information pertaining to the methodology of the CWB is available elsewhere (Aboriginal Affairs and Northern Development Canada, 2010).

#### 2.1.2 Water Risk

Using a request through *Access to Information*, we obtained First Nation communities’ drinking water risk ranking for 2006 from AANDC. The federal risk evaluation of First Nations drinking water systems is designed to help prioritize infrastructure projects and to develop long term strategies to minimize health risks.

The overall health risk ranking of a water system is based on a multi-barrier assessment using five categories: water source, system design, system operation and maintenance, operator training and certification, and record keeping and reporting. The weighting scheme is based on the assumption that while polluted water poses a high risk, the treatment facility is ultimately relied upon to deliver safe water (Indian and Northern Affairs Canada, Public Works and Government Service Canada, Environment Canada, Health Canada, 2005). Within each category there are several criteria that assessors evaluate to determine a risk ranking from 1 to 10. For example, the water source category risk criteria include the type of source water, availability of water, vulnerability of contamination, water quality, and source water protection. The system operation category considers the biological, chemical and physical standards, set by Health Canada, as an indication of the ability to provde safe drinking water. We provide an illustration of the Source Water category in Table 2. The risk evaluation guideline requires assessors to address additional considerations in determining the category risk ranking; however, the weighted overall risk level is the primary indicator of possible health threats (Table 3). A low risk indicates minor deficiencies; a medium risk system may pose a risk to human health, but is not an immediate concern; a high risk system requires immediate action to eliminate or minimize the human or environmental health threat (Indian and Northern Affairs Canada, Public Works and Government Service Canada, Environment Canada, Health Canada, 2005).

**Table 2:** Source water risk score calculation

Category	Criteria	Description	Risk Score
Source Water	Source	Groundwater	2

		Surface water	5

	Availability	Meets need	0

		Shortages now or with the last 5-10 years	1

		Does not meet demand	2

	Vulnerability to contamination	Unlikely	0

		Low	1

		Medium	2

		Multiple source	3

	Deteriorating water quality	Rending treatment ineffective	2

	Source water protection	No protection plan	2

		Plan designed for the community	0

**Table 3:** Calculating first nations overall water system risk score

Risk Evaluation Categories	Weighted Score (%)
Source Water	10
System Design	30
System Operation	30
Reporting	10
Operator	20

Overall Risk	Low (1-4)
	Medium (5-7)
	High (8-10)

Source: Indian and Northern Affairs Canada (Indian and Northern Affairs Canada, Public Works and Government Service Canada, Environment Canada, Health Canada, 2005).

### 2.2 Analysis

We used the First Nation community drinking water risk data and CWB (income, education, housing, labour force) to explore relationships. There were 739 First Nation drinking water systems assessed in 2006 that could be matched with 256 First Nations communities with CWB socioeconomic measures reported in 2006. Although there are several components to water quality, overall water quality risk (1-10) was analyzed in the current study.

Descriptive statistics were followed by correlation analysis, using three different measures of association: Spearman (*ρ*); Kendall’s tau (*τ*); and Pearson correlation (*r*). Pearson product-moment correlation is a parametric measure of the linear association between two variables while Spearman and Kendall’s tau correlations are non-parametric measures focused on the monotonicity of the relationship (Higgins, 2004). Differences between these measures served as a sensitivity analysis. All three measures have an associated magnitude and direction, ranging between -1 and +1, with no association being equivalent to a value of zero and higher values indicating a stronger relationship. For detailed information on assessing the relative strength of a correlation coefficient, see Cohen (1988). Confidence intervals (95%) were calculated for each measure of association.

Next, an unadjusted and adjusted ordinal regression (proportional or cumulative odds) model was run (Hosmer, Lemeshow, & Sturdivant, 2013). Overall water quality risk (1-10) was regressed on the CWB components (income, housing, labour force, education). All assumptions of the ordered regression model were assessed, including proportional odds. Coefficients from the regression model are presented as odds ratios for ease of interpretation. Statistically significant relationships are indicated (*p* < 0.05) along with 95% confidence intervals. All analyses were conducted with IBM SPSS Statistics 22.

## 3. Results

Descriptive statistics for the CWB and its components as well as the “Overall Water Risk Evaluation” are provided in Table 4.

**Table 4:** Descriptive statistics for the CWB and its components, and overall water risk evaluation

	Mean	SD	%	*n*
Community Well-being Index	0.543	0.098		256
Income	0.510	0.116		256
Education	0.318	0.121		256
Labour Force	0.684	0.083		256
Housing	0.660	0.147		256
Overall Water Risk Evaluation	4.637	1.696		256
Low (1-4)			54.3	139
Medium (5-7)			40.2	103
High (8-10)			5.5	14

As seen in Table 5, for the most part, the results showed no association between the CWB (and its components) and overall water risk, with the exception of labor force, which shows a weak (0.10-0.15) positive relationship. The findings were consistent across all three measures of association.

**Table 5:** Correlation between overall water Risk, and the CWB and its components

	Pearson’s *r*		Spearman’sρ		Kendall’sτ	

	Estimate	95% CI	Estimate	95% CI	Estimate	95% CI
Overall Risk						

CWB	.063	-.060, .191	.083	-.046, .206	.062	-.031, .152
Income	.058	-.050, .175	.082	-.047, .211	.065	-.028, .160
Education	.032	-.094, .168	.039	-.095, .166	.030	-.067, .124
Labour force	.142*	.018, .265	.131*	.001, .258	.099*	.004, .193
Housing	.014	-.103, .133	.010	-.108, .140	.009	-.075, .102

* *Note.* = *p* < 0.05;

** = *p* < 0.01; *n* = 256.

The ordinal regression model with all CWB components in the model was statistically significant (χ^2^ = 10.00, *p* = 0.04). Effect size was, however, low with Nagelkerke pseudo-*R*^2^ = 0.04, indicating a weak relationship between overall water risk and community socioeconomic indicators. Echoing the correlation analyses from Table 5, the results presented in Table 6 generally showed limited association between the CWB components and the overall risk assessment of water quality, with similar findings across the bivariate (unadjusted) and multivariable (adjusted) analyses. The only significant effect was labour force, with an adjusted odds ratio of 1.88 (*p* < 0.00), indicating a positive relationship with overall risk assessment of water quality.

**Table 6:** Unadjusted and adjusted odds ratios of overall water risk on CWB components (income, education, housing, and labour force)

	Unadjusted OR	Adjusted OR

	Estimate 95% CI	Estimate 95% CI
Overall Risk		

Income	1.12 0.92; 1.35	0.86 0.60; 1.20
Education	1.08 0.90; 1.29	0.90 0.70; 1.16
Housing	1.05 0.90; 1.22	0.99 0.80; 1.23
Labour force	1.45* 1.14; 1.85	1.88* 1.28; 2.76

* *Note.* = *p* < 0.00; *n* = 256

## 4. Discussion

This study sought to improve our understanding of access to safe drinking water across First Nation communities in Canada. Moreover, it is the first attempt to use national level data to generate a systematic understanding of the drivers underlying safe drinking water that inform decision making. Despite the importance in the literature of community level processes – particularly capacity – as a major social determinant of health and well-being (White, Beavon, & Spence, 2008; White, Maxim, & Beavon, 2004), in large part, there was no evidence of any meaningful relationships with safe drinking water across First Nations communities. In fact, the community capacity, as indicated by the CWB and three of its components (housing, income, education), failed to yield any insights into the processes regarding safe water, in contrast to arguments made elsewhere (White, Murphy, & Spence, 2012). These findings are, however, consistent with another rare nation-wide analysis that found no community level effects on health outcomes across reserve communities (Spence, 2015).

The one exception to this trend was the labour force component of the CWB, which, surprisingly, had a positive association with water risk. Of particular note, this relationship increased in magnitude between the unadjusted and adjusted ordinal regression. We caution that this finding may not indicate a true relationship; it could simply reflect the effects of other theoretically relevant determinants of safe water, such as geographic factors, which have played a role in related work (Spence, & Walters, 2012). Labour force participation and employment levels are associated with geographic factors, such as region and degree of isolation; for example, distance from major economic centres influences the size, diversity, and robustness of a community’s economy. Similarly, the relationship between geographic isolation and safe water has been documented elsewhere (Health Canada, 2009). Thus, geographic factors may be a logical confounder of the relationship observed in this study between labour force and safe water. At this point, it would seem diligent to reserve an exhaustive explanation of this unexpected relationship for future research when these findings are replicated, and coupled with consideration of confounding variables, such as geography.

As for the majority of null effects in this study, there are possible explanations that should be considered. First, the drinking water risk surveys are intended to provide a quick assessment of the main threats to human or environmental health. The validity and reliability of the survey results are questionable. A large number of community surveys have missing data or unanswered questions. The consultants administering the surveys spend two to three days gathering information about the water and wastewater facilities. In one case, the responses even appear to be conflicting; for the Dokis First Nation’s survey, the source water risk was given a medium risk (risk score = 7) because it was unknown whether the ground water directly connected to surface water. However, in the design section, the survey indicates that the ground water was not considered to be directly connected to surface water. This has implications on the type of drinking water treatment facility required for the community. Ground water under direct influence of surface water requires an advanced, more costly level of treatment.

Second, the level of analysis, community as operationalized by census sub-divisions, may be incorrect in terms of capturing processes influencing water risk. Similarly, determinants of safe water at other levels of analysis, such as the provincial or national level, could be more useful for understanding and intervening, as suggested elsewhere (White, Murphy, & Spence, 2012).

Third, the lack of association may indicate that other social processes at the community level are occurring, which are not captured by the CWB. Indeed, previous research has suggested that other central variables operating at this level of analysis, such as governance, cohesion, social capital, culture, and colonial practices, must be considered (White, Murphy, & Spence, 2012; von der Porten & de Loe, 2010; Reading et al., 2011). The availability of these measures was, however, a limiting factor in examining these issues in the current analysis.

## 5. Conclusion

Access to safe drinking water continues to be a major policy issue, particularly for vulnerable populations, including First Nations. This work contributes to the body of work on this issue, beyond small scale case studies and descriptive data, by demonstrating the null effects of processes at the community level, focused on key socioeconomic determinants, including income, education, labour force, and housing. Moving forward from a policy perspective, attention towards data quality are of critical importance, and scientifically, inquiry at theoretically relevant levels of analysis affecting safe drinking water are desperately needed. In summary, the complexity of safe water in First Nation communities across Canada is illustrated by this research. As a central determinant of health, access to safe drinking water must continue to be a research and policy priority within the broader goals of reducing social and health inequality between First Nations and the Canadian population.
